# Dataset for polyphonic sound event detection tasks in urban soundscapes: The synthetic polyphonic ambient sound source (SPASS) dataset

**DOI:** 10.1016/j.dib.2023.109552

**Published:** 2023-09-07

**Authors:** Rhoddy Viveros-Muñoz, Pablo Huijse, Victor Vargas, Diego Espejo, Victor Poblete, Jorge P. Arenas, Matthieu Vernier, Diego Vergara, Enrique Suárez

**Affiliations:** a Instituto de Acústica, Universidad Austral de Chile, General Lagos 2086, Valdivia, Chile; b Instituto de Informática, Universidad Austral de Chile, General Lagos 2086, Valdivia, Chile; c Millennium Institute of Astrophysics, Nuncio Monseñor Sotero Sanz 100, Providencia, Santiago, Chile

**Keywords:** Deep learning, Polyphonic sound event detection, Soundscape, Acoustic virtual reality

## Abstract

This paper presents the Synthetic Polyphonic Ambient Sound Source (SPASS) dataset, a publicly available synthetic polyphonic audio dataset. SPASS was designed to train deep neural networks effectively for polyphonic sound event detection (PSED) in urban soundscapes. SPASS contains synthetic recordings from five virtual environments: park, square, street, market, and waterfront. The data collection process consisted of the curation of different monophonic sound sources following a hierarchical class taxonomy, the configuration of the virtual environments with the RAVEN software library, the generation of all stimuli, and the processing of this data to create synthetic recordings of polyphonic sound events with their associated metadata. The dataset contains 5000 audio clips per environment, i.e., 25,000 stimuli of 10 s each, virtually recorded at a sampling rate of 44.1 kHz.

This effort is part of the project ``Integrated System for the Analysis of Environmental Sound Sources: FuSA System'' in the city of Valdivia, Chile, which aims to develop a system for detecting and classifying environmental sound sources through deep Artificial Neural Network (ANN) models.

Specifications Table

**Table:** 

Subject	Physical sciences: Acoustics and Ultrasonics

Specific subject area	Physical simulation of urban soundscapes to train deep learning models for polyphonic sound event detection.
Type of data	Digital audio files Metadata as CSV text files
How the data were acquired	Synthetic data were generated through acoustic virtual simulations using the room acoustics simulation framework RAVEN [1]. The SPASS dataset was built using multiple real monophonic sound examples. Those sounds were collected from public databases such as ESC-50 [2], UrbanSound8K [3], Making Sense of Sound dataset [4], Audio Event Net [5], and data from FreeSound web [6]. Then, the audio files were manually inspected and relabeled following a hierarchical urban sound event taxonomy.
Data format	Raw audio files in wav format Metadata in Comma-Separated Values (CVS) format
Description of data collection	Five urban soundscapes representing a market, street, park, plaza, and waterfront were simulated using the RAVEN software for virtual acoustic reality. A total of 25,000 10 s audio waveforms were generated. Each waveform simulates an omnidirectional microphone that records a set of randomly selected monophonic sound events convolved with (spatial) impulse responses from the simulated environments.
Data source location	• Institution: Universidad Austral de Chile • City/Town/Region: Valdivia city, region Los Ríos • Country: Chile ESC-50 https://github.com/karolpiczak/ESC-50 UrbanSound8K https://zenodo.org/record/1203745 Making Sense of Sound dataset https://figshare.com/articles/dataset/Making_Sense_Of_Sounds_Data_for_the_machine_learning_challenge_2018/6901475/1 Audio Event Net https://data.vision.ee.ethz.ch/cvl/ae_dataset FreeSound https://freesound.org/
Data accessibility	Repository name: Zenodo Data identification number: DOI:10.5281/zenodo.8239067 Direct URL to data: https://zenodo.org/record/8239067

## 1 Value of the Data

• SPASS is a high-quality synthetic dataset with perfect strong labels and sufficient data volume to effectively train large machine learning models for sound event detection. On the other hand, manually providing strong labels for large real audio datasets is a highly time-consuming, error-prone, and costly process.
• This data directly benefits researchers and engineers that require high-quality data to train a machine-learning model for urban sound event detection. There is also an indirect benefit to decision-makers and analysts using the models trained with SPASS to analyze urban soundscapes.
• SPASS was designed to pre-train a base model that can be fine-tuned to actual acoustics tasks related to urban soundscapes.
• The proposed methodology can synthesize a wide variety of soundscapes by providing an appropriate collection of monophonic sound events, e.g., natural, rural, and industrial soundscapes.
• The present dataset has potential use in transfer learning or fine-tuning processes with actual recordings of sound scenes (e.g., STARSS22, SINGA:PURA, or SONYC-UST) for the classification and detection of urban sounds.

## 2 Objective

To design and implement a methodology to synthesize a large polyphonic audio dataset with perfect strong labels to train machine learning models for urban sound event detection. This data article helps improve our experiments' reproducibility and may motivate other groups to continue building on this foundation.

## 3 Data Description

The FuSA system [7] taxonomy shown in Table 1 corresponds to the urban sound events that comprise the SPASS dataset. The hierarchical taxonomy considers seven coarse-level categories: Humans, Music, Animals, Environmental, Mechanics, Vehicles, and Alerts. The 33 fine-level categories can also be seen in Table 1.

**Table 1:** FuSA system's taxonomy for urban sound events.

Human	Music	Animals	Environmental	Mechanical	Vehicles	Alerts
talk	music	dog	rain	air_conditioner	car_idling	alarm
shouting		bird	river	cutting	car_moving	bells
crowd			water	drilling	motorcycle_idling	braking
steps			waves	explosives	motorcycle_moving	horn
			wind	fireworks	bus_idling	siren
				impact	bus_moving	
					truck_idling	
					truck_moving	
					Vwater	
					airborne	

The SPASS dataset was created from monophonic sound sources collected from public databases such as ESC-50 [2], UrbanSound8K [3], Making Sense of Sound dataset [4], Audio Event Net [5], and data from FreeSound web [6]. The audios were then manually inspected. Low-quality audio or audio that contained more than one sound source was discarded. Audios were also relabeled following the FuSA taxonomy. A total of one hundred monophonic sound sources were collected per class, except for the following classes:

• alarm=60,
• steps=35,
• bird=65,
• fireworks=43,
• motorcycle (idling=10, moving=33),
• car (idling=50, moving=5),
• bus (idling=6, moving=3),
• truck (idling=4, moving=19),
• air_conditioner = 84,
• Vwater=70,
• airborne=85, and
• braking=35.

Five environments were simulated: market, park, square, street, and waterfront. Each environment was built with a distinctive set of sound sources. The association between classes and environments is presented below:

• Market: talk, scream, crowd, steps, music, dog, birds, rain, wind, air conditioner, motorcycle, car, bus/truck, airborne, siren, alarm, and horn.
• Park: talk, scream, crowd, steps, music, dog, birds, rain, wind, water, fireworks, motorcycle, car, bus/truck, airborne, siren, horn, and bell.
• Square: talk, scream, crowd, steps, music, dog, birds, rain, wind, water, impact, cutting, digging, motorcycle, car, bus/truck, airborne, siren, alarm, horn, bell, and braking.
• Street: talk, scream, crowd, steps, music, dog, birds, rain, wind, impact, cutting, explosives, digging, air conditioner, motorcycle, car, bus/truck, airborne, siren, alarm, horn, and braking.
• Waterfront: talk, scream, crowd, steps, music, dog, birds, rain, wind, river, waves, impact, cutting, digging, fireworks, motorcycle, car, bus/truck, airborne, water vehicle, siren, alarm, horn, and braking.

The following section explains how the RAVEN software was configured to simulate these environments. A total of 5000 10 s audio clips were created for each environment. Consequently, the SPASS dataset consists of 25,000 polyphonic recordings containing diverse urban sound sources. All 10 s audios are mono channel, in 32-bit floating point WAV audio format, with a sampling rate of 44.1 kHz.

The metadata is a CSV table that includes the audio filename, taxonomic category, onset time (sec), offset time (sec), source location in xyz (m) coordinates, orientation of the recording microphone in xyz (m), and final location of moving sources in xyz (m). Note that the information ``source location in xyz(m)'' refers to the initial position of the moving sound sources. The information about the orientation of the recording microphone was meant for binaural recording, but for this mono-channel dataset, that information is irrelevant since the microphone is omnidirectional (Table 2).

**Table 2:** Metadata labels with examples.

Audio filename	Class	start (s)	end (s)	x (m)	y (m)	z (m)	Head orientation (xyz)			Final position of moving source (xyz)		
street00001.wav	talk	4.9	7.3	30.8	1.6	-15.4	-1	0	0			
street00001.wav	car_moving	3.8	10	16.4	1.0	-10.7	-1	0	0	77.6	1.0	-10.7
park00002.wav	music	1.1	6.1	63.3	1.6	-36.9	1	0	0			
park00003.wav	car_moving	1.5	7.8	12.3	1.0	-76.1	0	0	1	61.6	1.0	-76.1

The audio recordings are available in .7z format in the Zenodo open repository for download. The dataset consists of 5 audio folders (.7z format) containing all audio recordings, 5 files with the corresponding label of the audio folders (.csv format), and 5 files with the probability distribution of each sound event (.xlsx format).

**Fig. 1:**
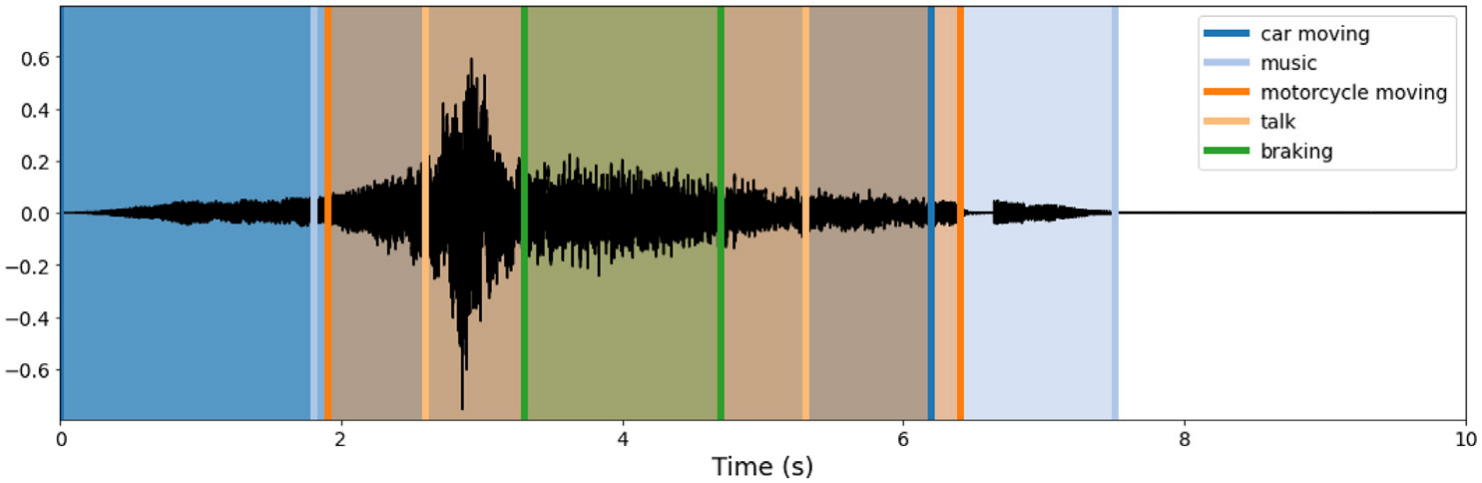
Audio data example.

Fig. 1 shows an example of the audio data with the labels superimposed. Each audio contains different sound events that are spread randomly in time and may overlap.

## 4 Experimental Design, Materials and Methods

All the monophonic sound sources were normalized ([−1, 1]) before simulation in the virtual scene. The location-related information was defined in Cartesian coordinates. Different sound absorption coefficients were defined depending on the material of the surfaces. These coefficients were taken from RAVEN´s database.

RAVEN (Room Acoustics for Virtual Environments) is a room acoustics simulation environment. It performs acoustic simulations in user-defined scenes where impulse responses (IR) can be generated for different positions within the simulated virtual environment. RAVEN is freely available for academic purposes [8].

Each of the five environments was designed differently. In what follows, we indicate the physical design of each environment:

• Market: A cube 80 (m) long, 50 (m) wide, and 80 (m) high, with a street at the end of one of the wide sides. Small buildings that simulate houses are added on the edge of both long sides. Buildings were considered as surfaces with different sound absorption coefficients following RAVEN´s material database. The floor and buildings were simulated as hard reflective surfaces. All other boundaries were simulated with a sound absorption coefficient close to one, thus creating a free-field acoustic environment.
• Park: A cube 80 (m) long, 80 (m) wide, and 80 (m) high, with a street at the end of one side. Open space with no buildings around. The floor was considered as acoustically soft floor similar to grass. All other boundaries were simulated with a sound absorption coefficient close to one, thus creating a free-field acoustic environment.
• Square: A cube 80 (m) long, 80 (m) wide, and 80 (m) high, with two opposite streets at the end of the wide sides. Open space with no buildings around. The floor was simulated as a hard reflective material such as concrete or paving stone. All other boundaries were simulated with a sound absorption coefficient close to one, thus creating a free-field acoustic environment.
• Street: A cube 80 (m) long, 20 (m) wide, and 80 (m) high, with two sidewalks around a street and buildings on the edge of both long sides. They were simulated as surfaces with different sound absorption coefficients following RAVEN´s material database. The floor and buildings were simulated as made of a hard reflective material. All other boundaries were simulated with a sound absorption coefficient close to one, thus creating a free-field acoustic environment.
• Waterfront: A cube 80 (m) long, 80 (m) wide, and 80 (m) high with a street at the end of the long sides and a river/wave area down the opposite side of the street. A pedestrian walkway was located between these two areas. The floor was considered to be made of a hard reflective material such as concrete. The river/waves surface was considered highly-reflecting since it is a characteristic of the interaction of a wave that travels through the air to water. All other boundaries were simulated with a sound absorption coefficient close to one, thus creating a free-field acoustic environment.

As mentioned in the previous section, every environment has its unique set of sound sources. Additionally, the probability of the appearance of each sound event in each environment was different. For example, the likelihood of finding a “dog” was higher in the park environment than a “braking.” The number of events of a given category and environment is randomly selected by drawing from a Poisson probability distribution with rate parameter (λ) specified in Table 3. The maximum number of occurrences is also limited, as shown in the table.

**Table 3:** Rate (λ) parameter of the Poisson probability distribution for each sound event. A symbol “-” indicates that the class is not considered for that category.

Class	Market		Park		Square		Street		Waterfront	
	λ	max	λ	max	λ	max	λ	max	λ	max
talk	2	3	2	3	2	3	1	2	2	2
Scream	0.35	1	0.25	1	0.25	1	0.35	1	0.25	1
Crowd	2	3	2	2	1.5	2	1.5	1	2	2
Steps	0.5	2	0.5	2	1	2	1.5	2	0.5	2
Music	2.5	2	1.5	2	1.5	2	1.2	2	1.5	2
Dog	0.5	1	2.5	5	1.5	4	1.5	2	2	3
Bird	0.5	2	2.5	6	1.5	4	1.5	2	2.5	4
Rain	0.5	1	0.5	1	0.5	1	0.5	1	0.5	1
River	–	–	–	–	–	–	–	–	2.5	1
Water	–	–	0.6	1	1	1	–	–	–	–
Waves	–	–	–	–	–	–	–	–	4	1
Wind	0.5	1	2	1	1	1	1	1	1	1
Air conditioner	1	2	–	–	–	–	0.5	2	–	–
Cutting	–	–	–	–	0.2	1	0.5	1	0.15	1
Digging	–	–	–	–	0.2	1	0.5	1	0.15	1
Explosives	–	–	–	–	–	–	0.1	1	–	–
Fireworks	–	–	0.25	1	–	–	–	–	0.25	1
Impact	–	–	–	–	0.2	1	0.5	1	0.15	1
Car	1	1	1	2	1.5	2	2	3	0.5	2
Bus/truck	0.5	1	0.5	1	1	1	1.5	1	0.5	1
Motorcycle	1	1	1	1	1	1	2	1	0.5	1
Water vehicle	–	–	–	–	–	–	–	–	0.8	2
Airborne	0.1	1	0.5	1	0.25	1	0.25	1	0.25	1
Alarm	0.25	1	–	–	0.25	1	0.25	1	0.15	1
Bell	–	–	0.5	1	0.5	1	–	–	–	–
Braking	–	–	–	–	0.25	1	0.25	1	0.15	1
Horns	0.25	1	0.5	2	0.5	2	1.5	2	0.25	1
Siren	0.25	1	0.25	1	0.25	1	0.25	1	0.15	1

A computer program was implemented in Matlab and interfaced with RAVEN to generate the Impulse Responses (IRs) for the virtual environments. The location of the IRs within the simulated geometry was selected randomly with a uniform distribution and the following additional constraints and assumptions:

• The sound events are located at least 2 m from the recording microphone and less than 1 m from the geometric boundaries of the scene.
• The motion simulations are limited to linear motions.
• An airborne vehicle is perceived as motionless from the recording point of view.

After randomly selecting a monophonic sound event and its location, the sound event is convoluted with the simulated IR. This process brings all the spatial characteristics to the monophonic signal. All monophonic signals are normalized. Therefore, the intensity of each sound event within the simulated environment depends solely on its distance from the virtual recorder. The convolved sound signal is then randomly inserted into a 10 s container waveform, nevertheless, the selection was drawn from a uniform distribution on [0,10-t], where t is the duration (in seconds) of the sound signal. The start and end times and the spatial position of each sound event are automatically recorded in Cartesian coordinates in the metadata file. This process is repeated according to the probability of occurrence of each sound event, which in turn is associated with the taxonomic class. This procedure results in a polyphonic audio file with start and end time tags of all included sound events and their location in space.

## Ethics Statements

This work did not include research on humans, animals, or data collected from social media platforms. We confirmed that the data distribution policies of the primary data sources used to construct SPASS were complied with.

## CRediT authorship contribution statement

**Rhoddy Viveros-Muñoz:** Conceptualization, Formal analysis, Data curation, Methodology, Investigation, Visualization, Software, Validation, Writing – original draft. **Pablo Huijse:** Conceptualization, Software, Methodology, Investigation, Formal analysis, Validation, Writing – original draft, Writing – review & editing. **Victor Vargas:** Visualization, Software, Data curation. **Diego Espejo:** Visualization, Software, Data curation. **Victor Poblete:** Investigation, Visualization, Validation, Software, Data curation, Supervision. **Jorge P. Arenas:** Resources, Supervision, Project administration, Writing – review & editing. **Matthieu Vernier:** Methodology, Software. **Diego Vergara:** Resources. **Enrique Suárez:** Funding acquisition, Supervision, Validation, Project administration.

## Data Availability

SPASS dataset (Original data) (Zenodo).
